# Management Options for Extensor Mechanism Discontinuity in Patients With Total Knee Arthroplasty

**DOI:** 10.7759/cureus.9225

**Published:** 2020-07-16

**Authors:** Parth Vyas, Quanjun Cui

**Affiliations:** 1 Orthopedic Surgery, University of North Dakota/Sanford Health, Fargo, USA; 2 Orthopedic Surgery, University of Virginia Health System, Charlottesville, USA

**Keywords:** knee, extensor mechanism, knee replacement, patellar tendon rupture, discontinuity, total knee replacement

## Abstract

Extensor mechanism disruption is one of the most dreaded complications of total knee arthroplasty. At times, the disruption is associated with infection, the paucity of soft tissue, and loosening of implants. Treatment decisions made by surgeons are guided by their experience and expertise. The purpose of this article is to provide the readers with an evidence-based comprehensive review which, in turn, should help them in diagnosis and selecting the best treatment strategy for individual patients.

In the following article, we have discussed extensor mechanism disruptions of varying severity at various anatomical levels. We also covered both operative and non-operative measures in different clinical situations.

The analysis of various articles published in the literature would also help orthopedic surgeons to understand the probable outcomes of the particular treatment option chosen and to counsel their patients accordingly.

## Introduction and background

The extensor mechanism of the knee joint is one of the most important structures encountered during total knee arthroplasty. It consists of the quadriceps tendon, patella, and patellar tendon. The importance of extensor mechanism is well-established in the field of knee replacement, not only in terms of minimizing intraoperative complications but also in early postoperative recovery and in attaining long-term goals. The structural and functional integrity of the extensor mechanism is crucial for optimum biomechanics of both the native and prosthetic knee. Restoration of the optimum Q-angle is necessary to obtain desirable patellar tracking and to minimize the chances of anterior knee pain [1]. 

One of the major steps during any knee replacement surgery is to retract the extensor mechanism in a way that minimizes damage to the structural and functional integrity of the extensor mechanism and, at the same time, to obtain adequate exposure to performing all the necessary procedures. All the contemporary approaches to the knee joint revolve around how to handle the extensor mechanism during the surgery and how to ensure the optimum function of the extensor mechanism postoperatively. The rationale presented by surgeons using subvastus and midvastus approaches is that they are less damaging to the extensor mechanism and hence help in faster recovery [2].

For revision cases, the biggest obstacle in attaining proper exposure is stiff and sometimes deficient extensor mechanism. Extensive approaches like the rectus snip, V-Y quadricepsplasty, and tibial tubercle osteotomy have been described in the literature for such cases [3]. These extensile approaches help the surgeon to obtain adequate exposure in performing such complex procedures.

Extensor mechanism dysfunction in the recipients of total knee arthroplasty can result from various causes ranging from iatrogenic to post-traumatic disruption. This dysfunction can be acute, subacute, or chronic and can involve any of the anatomical components of the extensor mechanism. Sometimes extensor mechanism disruption is combined with other complications, such as infection, periprosthetic fracture, loose implants, and failed previous reconstructions. These coexisting conditions have profound implications on the management and outcomes of extensor mechanism repair. Adequate repair or reconstruction of the extensor mechanism is considered paramount for the success of knee replacement in such cases.

Based on all the aforementioned factors, there are numerous management strategies described in the literature to treat this rare but devastating complication [1-35]. To date, there is no consensus amongst orthopedists regarding ideal treatment for extensor mechanism deficiency, especially in chronic cases. Treatment decisions made by surgeons largely depend upon individual training, experience, and preferences rather than evidence-based guidelines.

The purpose of this review is to summarize the existing literature regarding extensor mechanism reconstruction and provide the readers an evidence-based summary of etiology, diagnosis, and techniques for extensor mechanism reconstruction and outcomes of various strategies to treat this catastrophic condition.

## Review

Material and methods

We searched the PubMed database using "extensor mechanism disruption" and "total knee arthroplasty (TKA)" as keywords. Resultant articles were filtered based on human subjects, English language, and clinical studies. All the remaining articles were manually scanned by the authors for their relevance to the subject. The resultant 35 articles were thoroughly reviewed for their entire content and classifications; etiology and management portions of the articles were derived based on the evidence portrayed in the selected articles.

Classification

**Figure 1:**
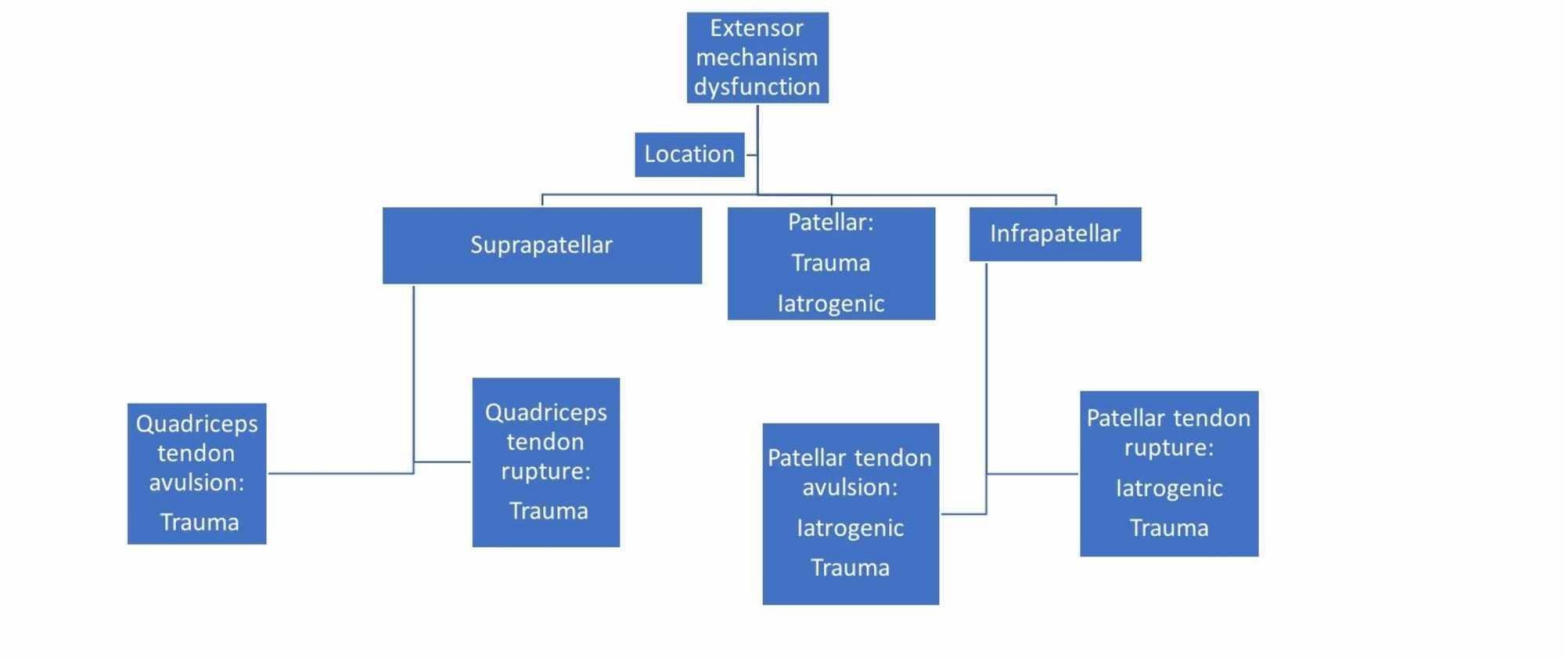
Anatomical classification of extensor mechanism disruption

Extensor mechanism disruptions can be further categorized based upon location, extent, or chronicity of the disruption. Extensor mechanism disruption can occur at the level of the quadriceps tendon, patella, or patellar tendon, and management options may vary accordingly (Figure 1).

Patellar tendon disruptions are the most common and the most devastating form of extensor mechanism dysfunction, mainly because of the poor soft tissue coverage, lack of adequate blood supply, and resultant extremely high incidence of failures and considerable reoperation rates [4]. 

Etiology

The etiology of the dysfunction has a great influence on the management and outcomes. Acute traumatic disruptions are relatively easier to treat since scarring and retraction of the extensor mechanism is minimal and primary repair is still an option in many such cases, while chronic or failed reconstructions are often associated with infection or loosening of components. These cases are less amenable to primary repair, and failure rates and complications are higher.

Suprapatellar disruptions result from either a quadriceps tendon rupture or avulsion from the superior pole of the patella. It is a rare complication in comparison to patellar tendon rupture or avulsion. The estimated incidence is 0.1% amongst total knee replacement recipients [5]. While trauma is often considered as a trigger event, there are some predisposing factors that increase the risk of rupture in individuals (Table 1) [6-8].

**Table 1:** Risk Factors for Quadriceps Tendon Rupture

Systemic Risk Factors	Local Risk Factors
Rheumatoid arthritis	Prior knee arthroplasty
Diabetes mellitus	Arthrotomy
Chronic renal failure	Multiple steroid injections
Obesity	Patellar malpositioning
Hyperthyroidism	Lateral retinacular release at the time of the arthroplasty

Periprosthetic fracture of the patella is the second most common periprosthetic fracture around the knee joint after distal femur fracture [9]. The estimated average incidence is 1.19% [10]. It is one of the most difficult fractures to treat. It is worth noting that only about 12% of these fractures are associated with trauma [11]. Most of these present at routine follow-up with anterior knee pain with or without extensor lag. Most of these fractures are the result of osteonecrosis of the patella. The etiology of almost all of these fractures is iatrogenic.

There are some risk factors associated with periprosthetic patella fracture. The fact that 99% of these fractures happen in a resurfaced patella shows that resurfacing of the patella is a prominent risk factor. This phenomenon challenges the wisdom behind the practice of resurfacing every patella routinely during primary TKA. The residual thickness of the patella is a known contributor to the risk of fracture. Traditionally, at least 12 mm of the thickness of the residual patella is considered adequate to resurface the patella. This signifies the importance of measuring the thickness of the patella before proceeding with a bony resection. A lateral release to improve patella tracking is detrimental to the blood supply to the patella. This becomes more important in the setting of arthroplasty where the medial parapatellar arthrotomy has already jeopardized the medial blood supply. An additional release laterally can render the patella completely avascular and results in osteonecrosis of the patella and resultant stress fracture. All care must be taken to identify and protect the superior lateral genicular artery while performing lateral release. For reasons not clearly understood, cementless components are associated with an increased incidence of fractures. The components with a large central peg create a stress riser and increase the risk of fracture [10-11]. Component malalignment alters the biomechanics of the knee joint, increases the stress on the prosthetic patellar component, and indirectly increases the chances of fracture [12-13].

These fractures most commonly occur in the first two years after surgery with an average time period being 18.5 months [10].

Ortiguerra and Berry classified periprosthetic patella fracture in three types. Their classification is based on the integrity of the extensor mechanism, the presence or absence of loosening of the component, and the remaining bone stock [10, 13-14]. This classification, with the relative incidence of individual types of fractures, is depicted in Table 2.

**Table 2:** Ortiguerra and Berry Classification of Periprosthetic Patellar Fracture [14]

Type	Description
Type I	Intact extensor mechanism and stable implant (25.3%)
Type II	Disruption of extensor mechanism with or without implant in place (20%)
Type III	Intact extensor mechanism and loosening of the patellar component (54.7%)
A	Reasonable remaining bone stock
B	Poor bone stock

Most of the patellar tendon avulsions or ruptures can be considered iatrogenic, resulting from either direct injury during the surgery or component malalignment putting excessive stress on the patellar tendon during successive ambulation [15]. Patellar tendon rupture or avulsion is one of the most dreaded complications associated with total knee arthroplasty. Regardless of the treatment modality used, the outcomes are unsatisfactory. To prevent these injuries, it is important to prevent excessive stress on the patellar tendon and, if needed, to perform adequate arthrolysis or to switch to extensile approaches, like V-Y quadricepsplasty or rectus snip, at the appropriate time to prevent rupture or avulsion of the patellar tendon [16]. In some instances, nonunion of the tibial tubercle osteotomy used as an extensile approach can lead to patellar tendon insufficiency.

Diagnosis

**Figure 2:**
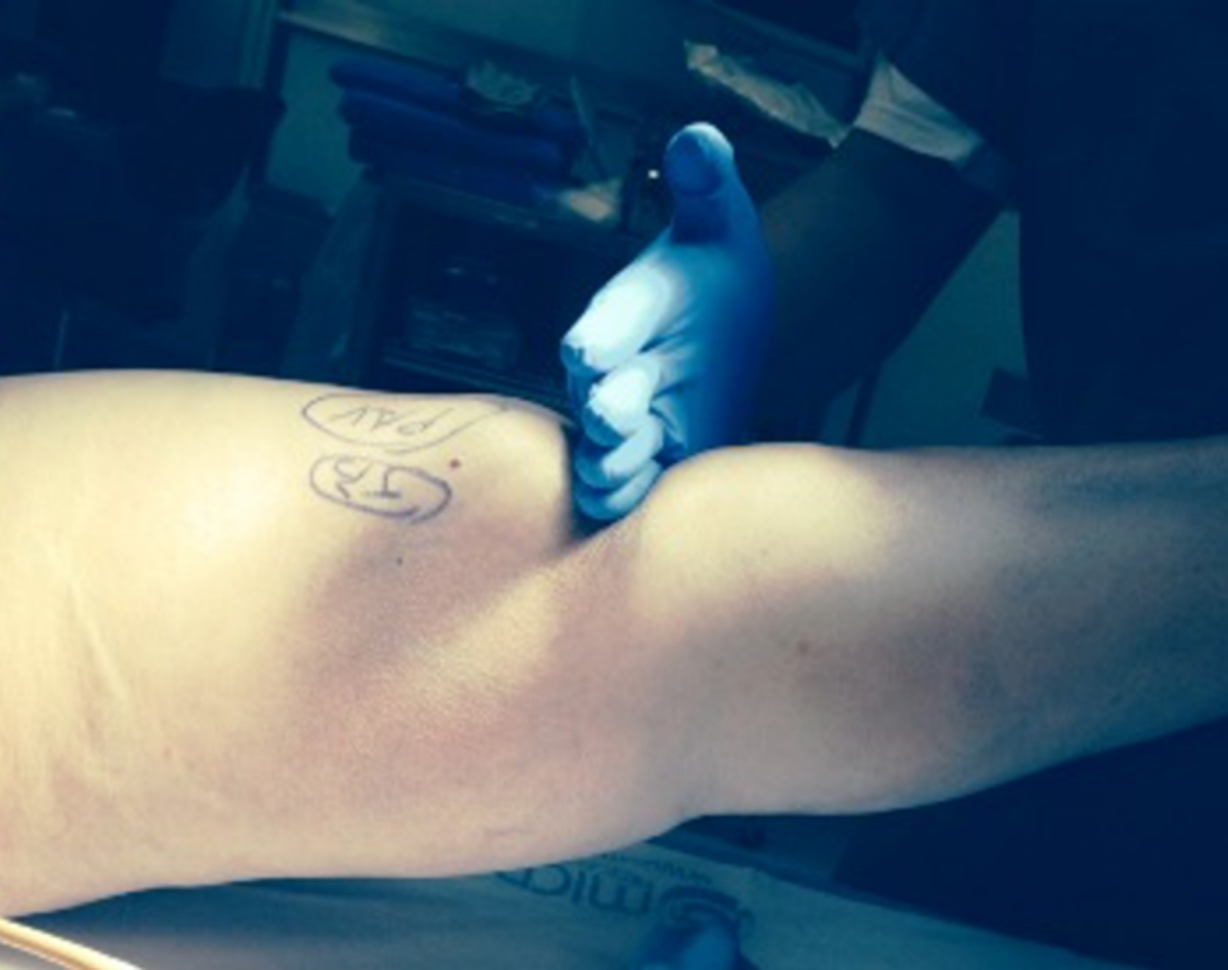
Palpable defect in the infrapatellar region at the site of patellar tendon disruption

Regardless of the etiology and anatomic location, the diagnosis is mainly clinical. In many cases, the diagnosis is delayed or missed altogether due to a lack of suspicion. Patients with extensor mechanism dysfunction typically present with loss of active extension, instability, and quadriceps minus gait. Patients have difficulty in climbing stairs. A history of trauma is commonly present but should not be overemphasized since, in many cases, it cannot be directly linked to the clinical picture. Anterior knee pain is a common presentation in patients with patella fractures with or without extensor lag, depending upon the degree of displacement and continuity of the extensor mechanism. In many cases, especially with complete disruption of the extensor mechanism, a palpable defect virtually confirms the diagnosis (Figure 2).

In cases of a complete patellar tendon disruption, the patella alta is also an important clinical finding.

Apart from the degree of extensor lag and instability, a thorough history and clinical examination also help in determining the future surgical approach, availability of local soft tissue and flaps, the blood supply to the region, the presence or absence of infection, the necessity of additional procedures (e.g., revision of one or more components), medical comorbidities, and probable outcomes.

The clinical diagnosis can be further validated by appropriate radiological studies. A plain x-ray is usually sufficient in this regard. Direct evidence of extensor mechanism disruption can be seen on plain x-ray in the form of patella alta (patellar tendon disruption), tibial tuberosity avulsion, patellar fracture, nonunion or displacement of tibial tubercle osteotomy, or an anteriorly displaced patella (quadriceps tendon disruption). Imaging studies confirm the diagnosis and provide important information about component alignment, fixation of the component, and remaining bone stock. Magnetic resonance imaging (MRI) with special sequences or ultrasound may be helpful in some selected cases but are not routinely required.

The optimum treatment strategy is devised based on all the inputs from the patient's history, physical examination, and imaging studies. The final treatment strategy not only depends upon location and degree of extensor mechanism disruption but also on preexisting medical conditions, previous attempts at reconstructions, and expected overall outcomes.

Management

Quadriceps Tendon Rupture or Avulsion

Incomplete ruptures can be treated non-operatively with acceptable results. In patients with less than a 20-degree extensor lag, incomplete ruptures can be treated with immobilization of the knee in extension for four to six weeks, either in a cast or in a brace, followed by gradual mobilization and physical therapy [5, 17]. A study done by Dobbs et al. showed that 85% of cases with partial quadriceps tendon ruptures yielded satisfactory outcomes with non-operative treatment. This is in contrast to the operative treatment of partial ruptures, which yields satisfactory outcomes in about 75% of cases. The same study shows satisfactory outcomes in only 40% of cases with complete ruptures. These results signify the importance of adequately protecting partial ruptures and thus preventing them from being converted to complete ruptures [5].

When extensor lag is more than 20 degrees, operative intervention is necessary. For acute ruptures, primary repair following thorough debridement of the severed ends is recommended. Typically, Krackow sutures are placed in the quadriceps tendon and it is attached to the patella using either drill holes or suture anchor. Biomechanically suture anchor repairs are considered more robust [18]. For chronic ruptures, the primary repair is often impossible owing to the contracture of the surrounding tissue and retraction of the extensor mechanism. In such cases, autologous augmentation using the vastus medialis, vastus lateralis, and medial head of the gastrocnemius can be used [4, 18]. In most cases, local augmentation is not sufficient because of the compromised soft tissue envelope and vascularity. However, it is proven that when a repair is augmented with the medial head of the gastrocnemius with or without the medial part of the soleus extensor lag is less pronounced in comparison to vastus medialis and lateralis flap advancement alone [5]. Results reported by Dobbs et al. are discouraging with a 35% reoperation rate and 12% infection rate. Forty percent of the primary suture repairs had re-ruptured [5]. These results are in stark contrast with the generally good results reported in patients without knee replacement [20]. These differences can probably be explained by a compromised soft tissue envelope, higher infection rates, comorbidities, and a relatively older patient population in knee replacement recipients. Chronic and severe deficiency of the quadriceps tendon can be managed by an Achilles tendon allograft with calcaneal tuberosity bone block, extensor mechanism allograft, or patellar tendon allograft. Recently, the use of Marlex mesh has also been popular, which is discussed in detail in the section of patellar tendon disruptions. Results of operative interventions of chronic rupture and resulting insufficiency are far less than satisfactory. 

Even after surgical repair, immobilization in extension for a period of four to six weeks, followed by gradual mobilization, is recommended to protect the repair.

Patellar Fracture

**Figure 3:**
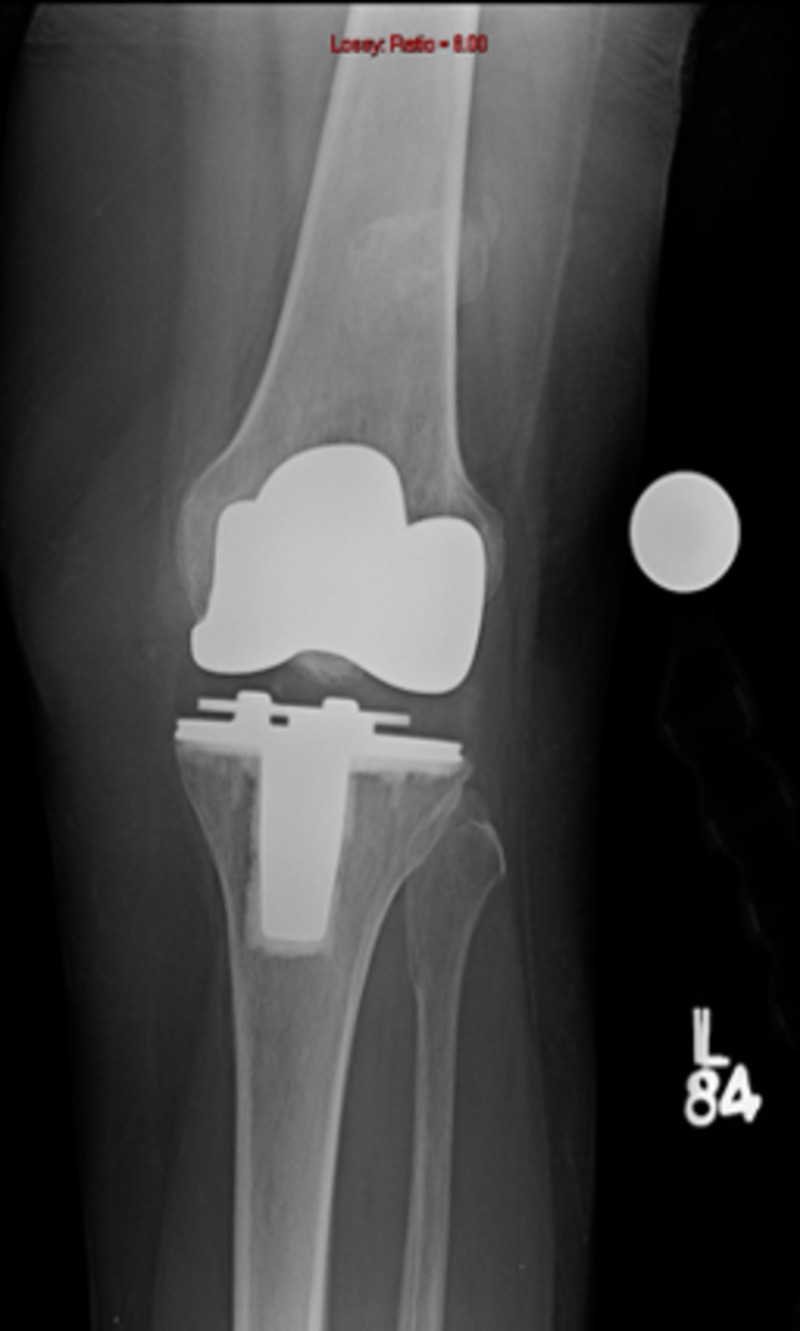
Periprosthetic patellar fracture, anteroposterior view

**Figure 4:**
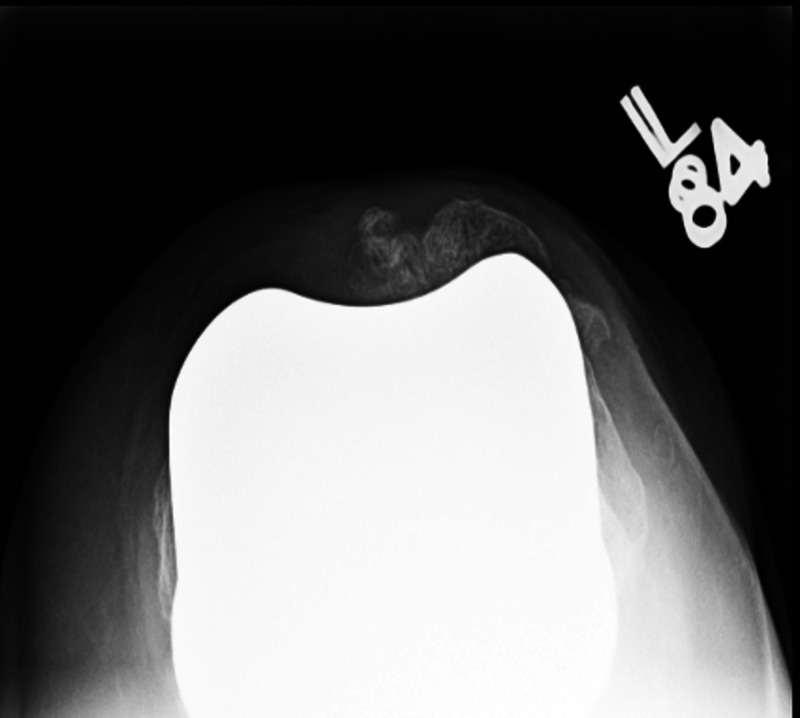
Periprosthetic patellar fracture, merchant view

**Figure 5:**
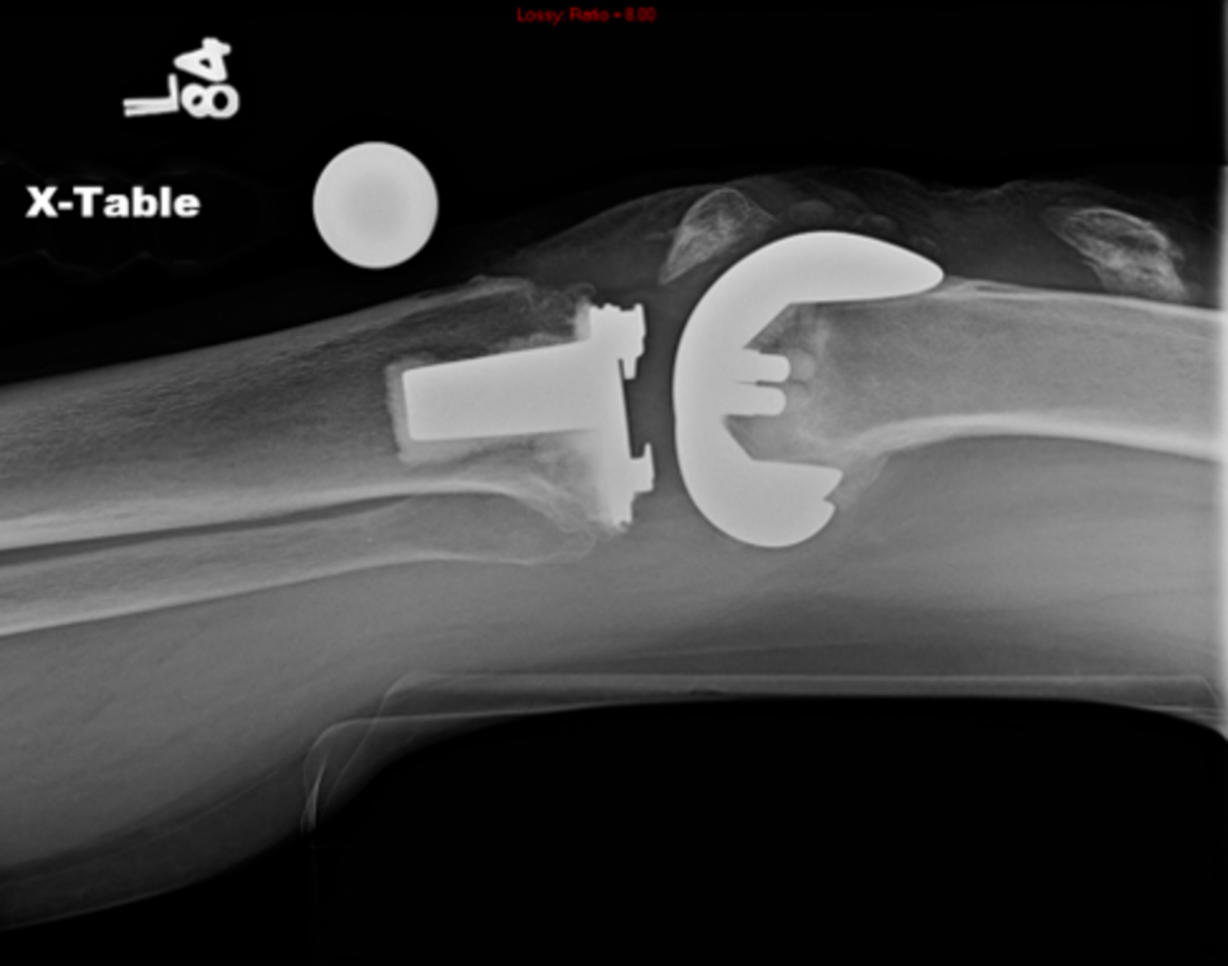
Periprosthetic patellar fracture, lateral view

While considering various treatment options, it should be clearly understood that these fractures are different than traumatic patellar fractures in a native knee. Outcomes of open reduction and internal fixation (ORIF) are poor in these patients [10]. Reasons for this discrepancy in results lie in the fact that these fractures occur in the background of poor vascularity, low residual bone stock, and altered biomechanics (Figures 3-5).

Patients with acceptable quadriceps function and well-fixed components can be treated conservatively in the same manner as a quadriceps tendon rupture [10]. Non-operative treatment yields satisfactory results in most patients as per Keating et al. [11]. Mean flexion achieved was around 120 degrees with less than 5 degrees of extensor lag and minimal pain in most of their patients.

**Figure 6:**
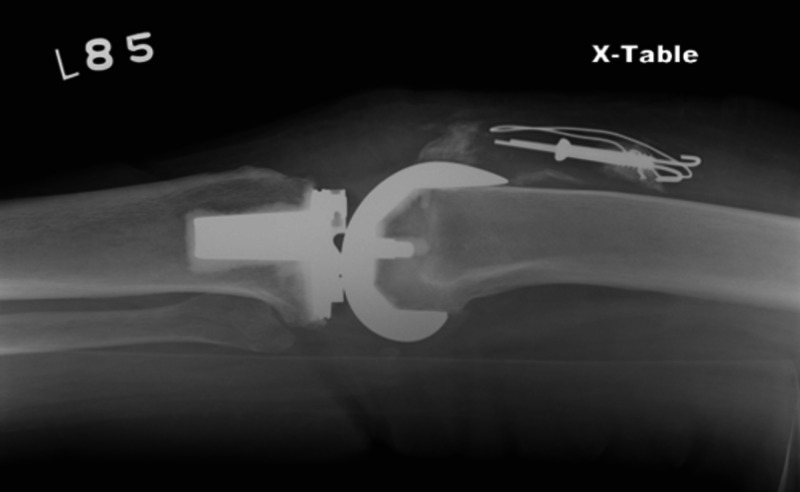
Failed open reduction and internal fixation performed for periprosthetic patellar fracture (lateral view)

**Figure 7:**
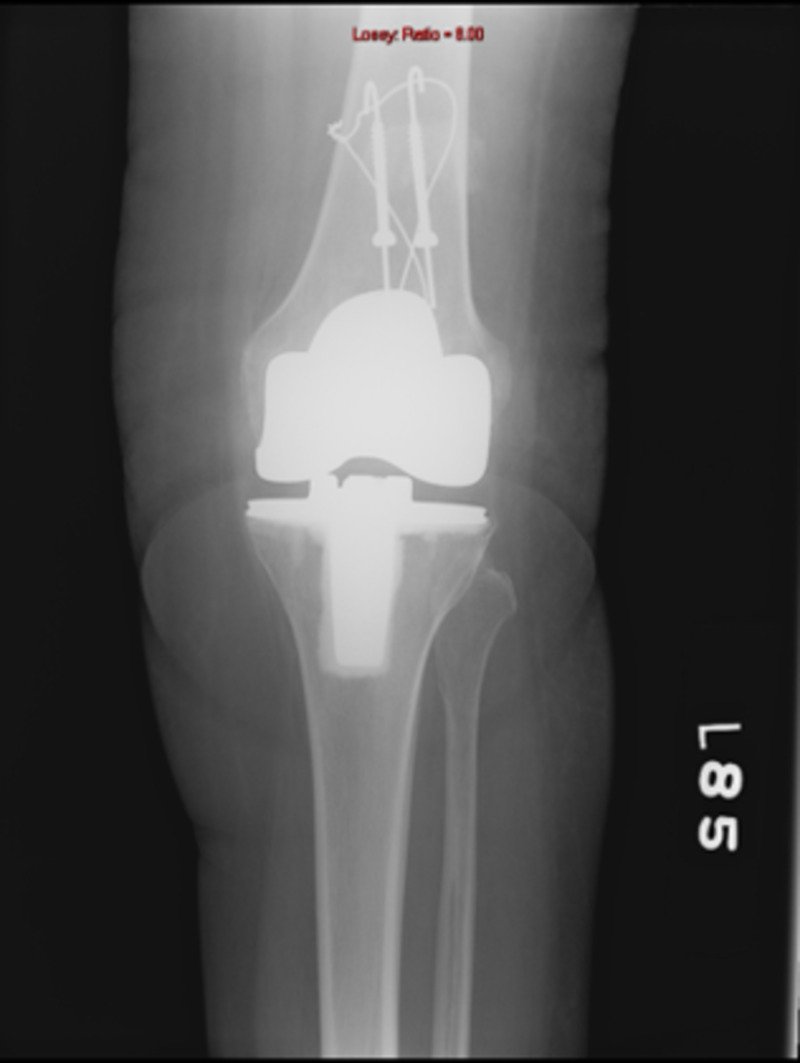
Anteroposterior view

Operative treatment is associated with a guarded prognosis. ORIF is associated with an 88% failure rate (Figures 6-7) [10].

This fact signifies the basic difference between a periprosthetic fracture of the patella and traumatic fractures of the native patella. This high failure rate is related to poor bone stock and vascularity. The high failure rate, in addition to the potential of hardware-related complications like breakage and prominent hardware, makes ORIF an inappropriate choice. Partial excision of the patellar fragment with or without repair of the extensor mechanism, as needed, is the mainstay of the operative treatment of patients with periprosthetic patellar who are symptomatic. In cases of a resurfaced patella, integrity and tracking of the patellar prosthesis should be addressed in addition to the extensor mechanism reconstruction. Whenever the patellar component is loose, it should be removed. Further reconstruction is based on available bone stock and the quality of the remaining bone. If an adequate bone stock is available (at least 12 mm), patellar resurfacing may be a viable option. Nowadays, with some newer designs (e.g., trabecular metal components), resurfacing may be considered in selected cases with lower bone stock [10].

Patellar Tendon Rupture or Avulsion

Partial disruptions with less than 30 degrees of extensor lag can be treated non-surgically in the same line as described in previous sections. Patients with more significant extensor lag should be treated surgically, provided there are no contraindications [17].

Surgical repair of this condition is extremely challenging because of the constant pull on the repair by the extensor mechanism and inadequate soft tissue coverage available locally.

Direct repair almost always fails and leads to unacceptable outcomes. The main reason for failure is the paucity of soft tissue to achieve adequate strength to withstand the forces during ambulation, which are concentrated on a relatively small area. The vascularity of the tendon is usually poor due to repetitive parapatellar arthrotomies performed in the past. In many cases, there is component malalignment responsible for excessive stress on the extensor mechanism. For these reasons, direct repair is rarely performed nowadays for chronic disruptions and some sort of augmentation is usually considered. Acute patellar tendon avulsions can be treated with either soft tissue repair or direct repair to the bone using drill holes or suture anchors [21]. Acute mid-substance tears can be treated with end-to-end repair, usually augmented by the semimembranosus, gracilis, or Achilles tendon. Table 3 depicts the studies related to local flap augmentation for extensor mechanism disruptions.

**Table 3:** Results of Local Soft Tissue Augmentation for Extensor Mechanism Reconstruction HSS: Hospital for Special Surgery; N: number

Study	Technique	N	Results	Conclusion
Whiteside et al. [22]	Vastus medialis (VM) and vastus lateralis (VL) flap reconstruction with or without soleus or gastrocnemius (GN)	8	Mean extension lag 22° (5° - 65°); lag is less when gastrocnemius or soleus transfer was performed	VM and VL flaps can cover anterior defects but extensor lag remains an issue unless GN and/or soleus are also transferred.
Whiteside et al. [23]	Vastus medialis (VM) and vastus lateralis (VL) flap reconstruction with medial gastrocnemius (GN) flap for extensive defects closed by failed allograft reconstruction	5	Mean extensor lag: 47° preoperative to 12° postoperative at 1 year	Medial GN flap, in addition to VM and VL flaps, provide a robust site for tendon reconstruction in cases of failed allograft reconstruction
Busfield et al. [24]	Extended GN flap reconstruction for extensor mechanism loss with (N:7) or without (N:2) arthroplasty	9	Average lag: 13.5°. Independent ambulation in all patients	GN flap can provide adequate coverage with reasonable outcomes and may alleviate the need for an allograft
Cadambi et al. [25]	Semitendinosus muscle autograft	7	Average extensor lag: 8.7°. Average improvement in HSS Knee Society score: 72 postoperatively	This technique can restore function and is better than direct repair and suture anchor reconstruction

**Figure 8:**
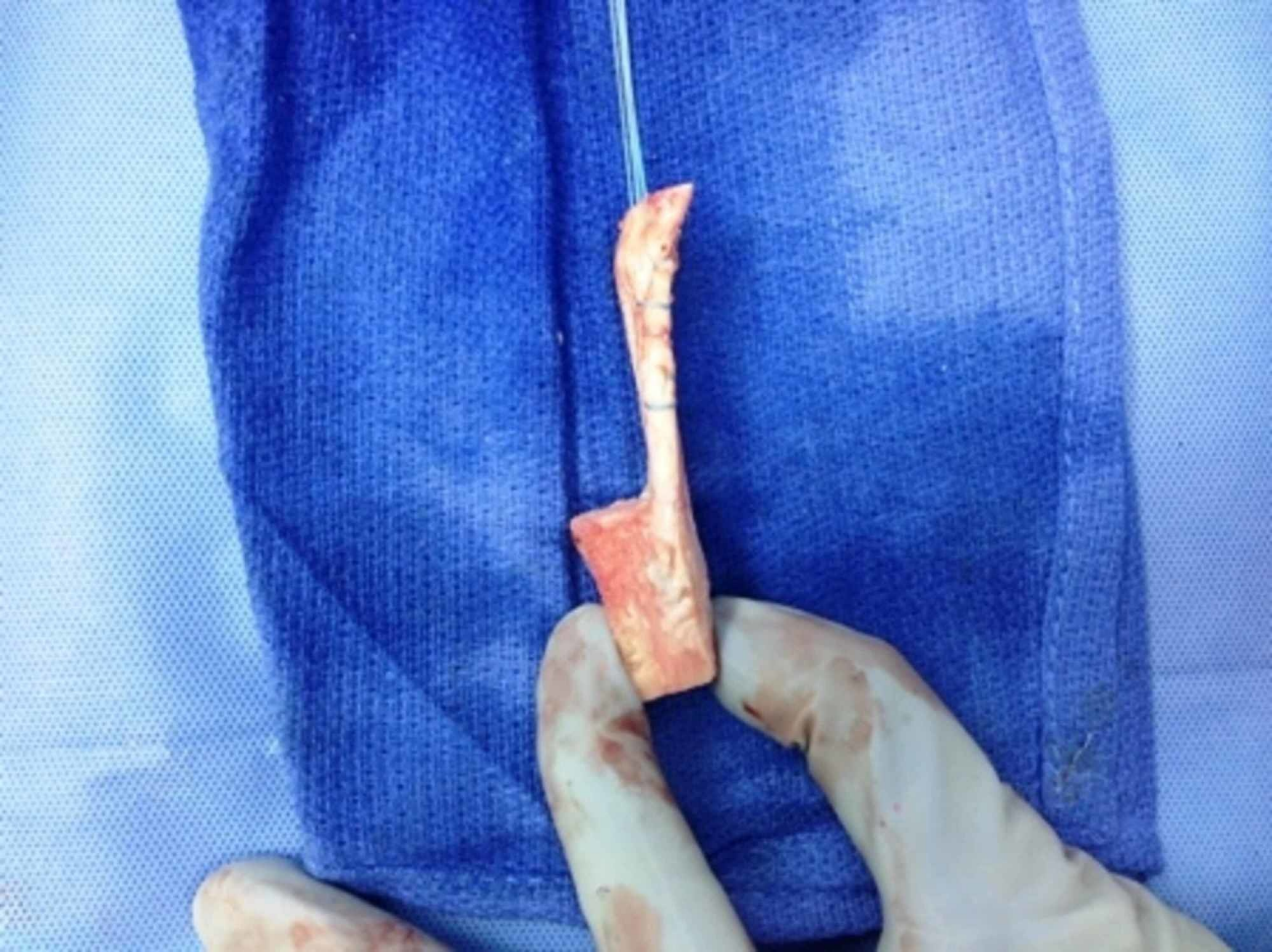
Patellar tendon-bone allograft with Krackow sutures passed through the patellar tendon

**Figure 9:**
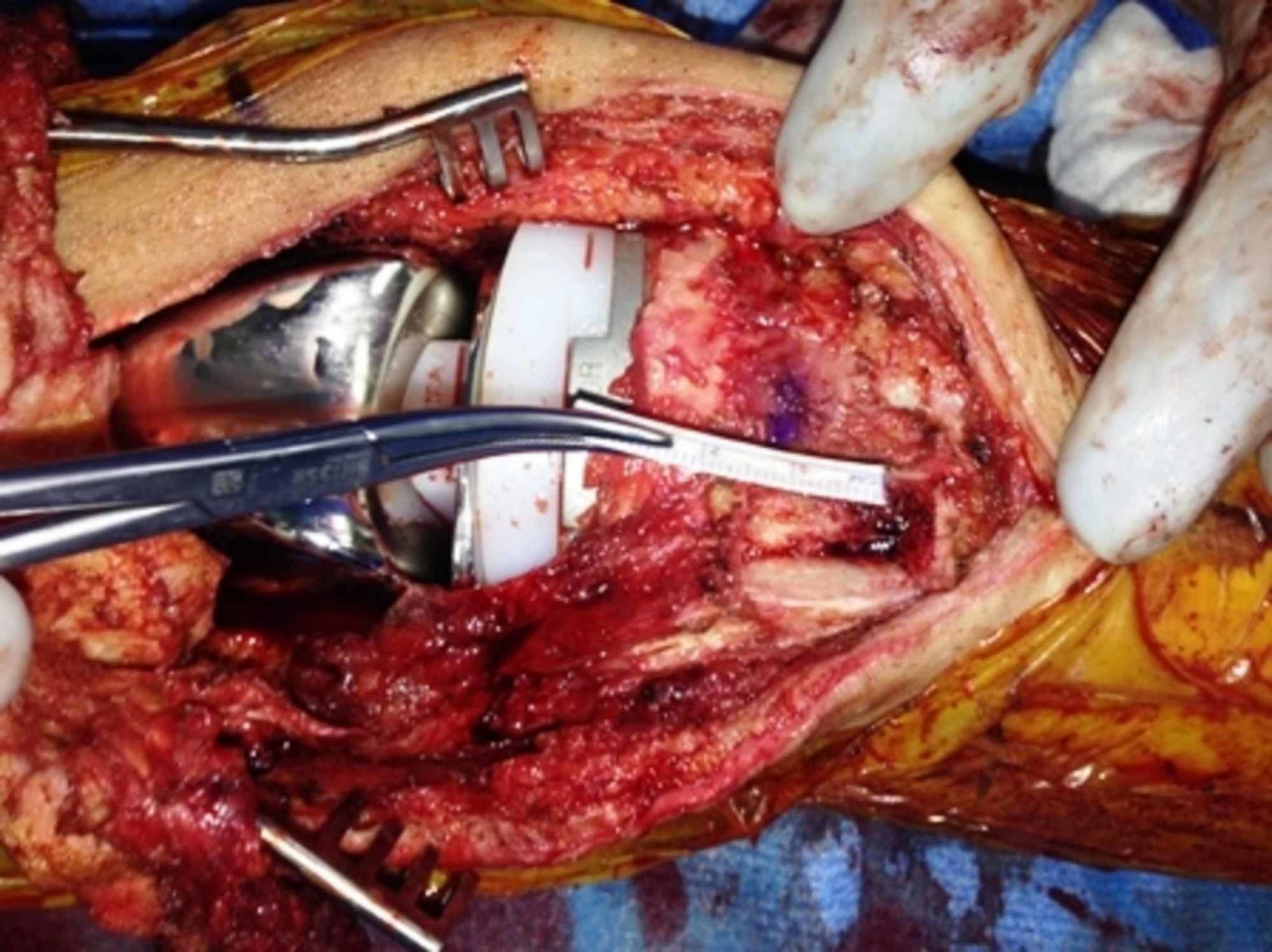
Trough in the proximal tibia for fixation of the allograft bone block

**Figure 10:**
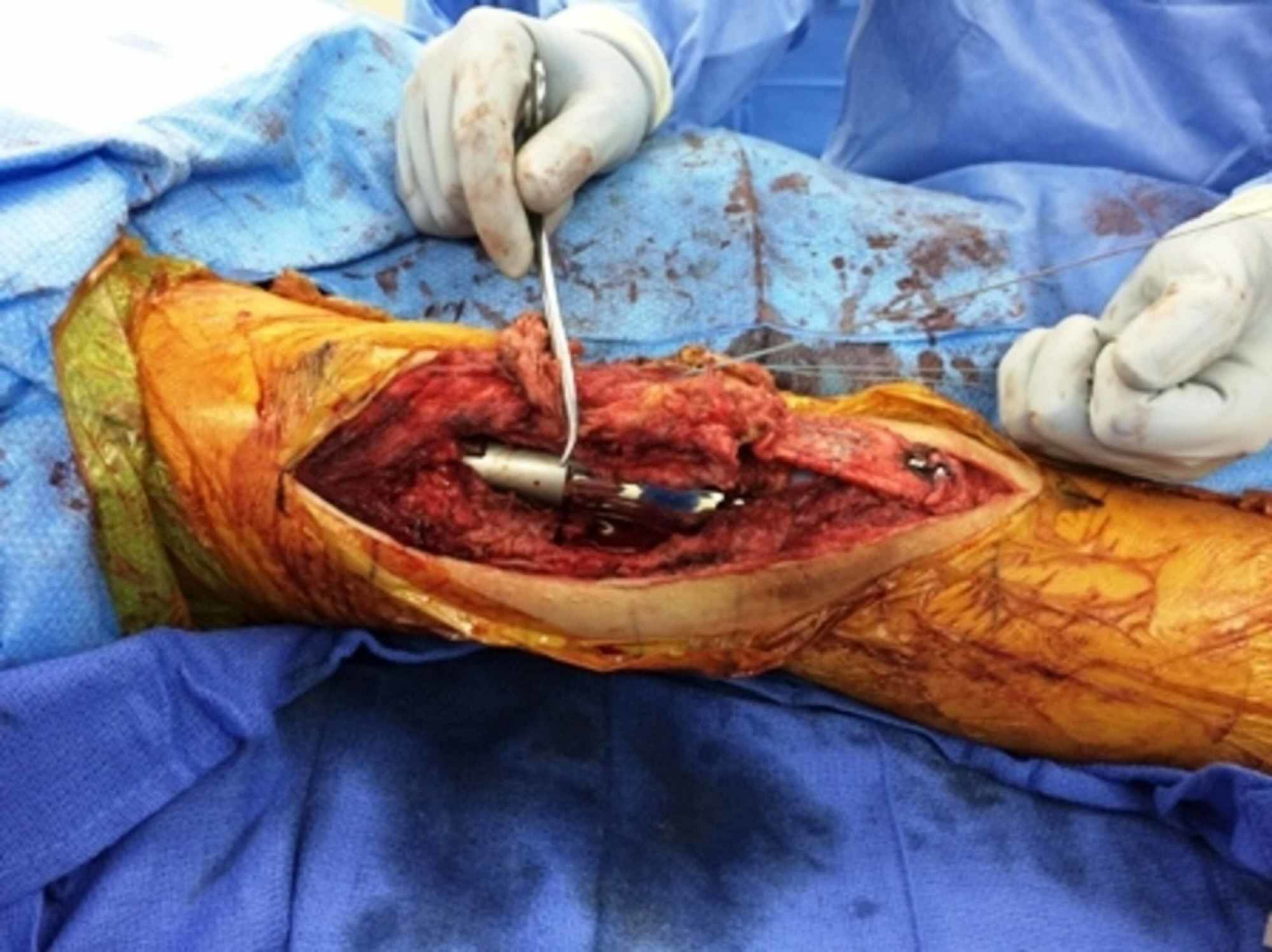
Fixation of the allograft bone block to the tibia using two 3.5 mm screws; attachment of the allograft tendon to the lower pole of the patella through the bone tunnel using FiberWire® sutures (Arthrex, Inc., Naples, FL)

**Figure 11:**
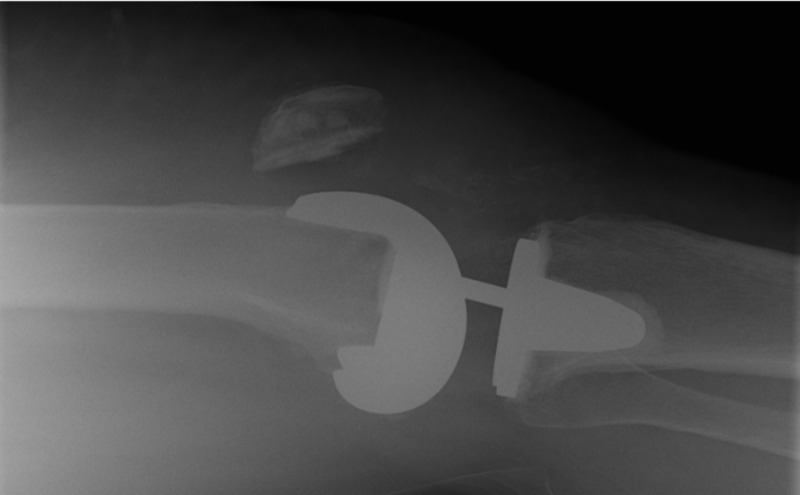
Preoperative lateral x-ray showing a patella alta

**Figure 12:**
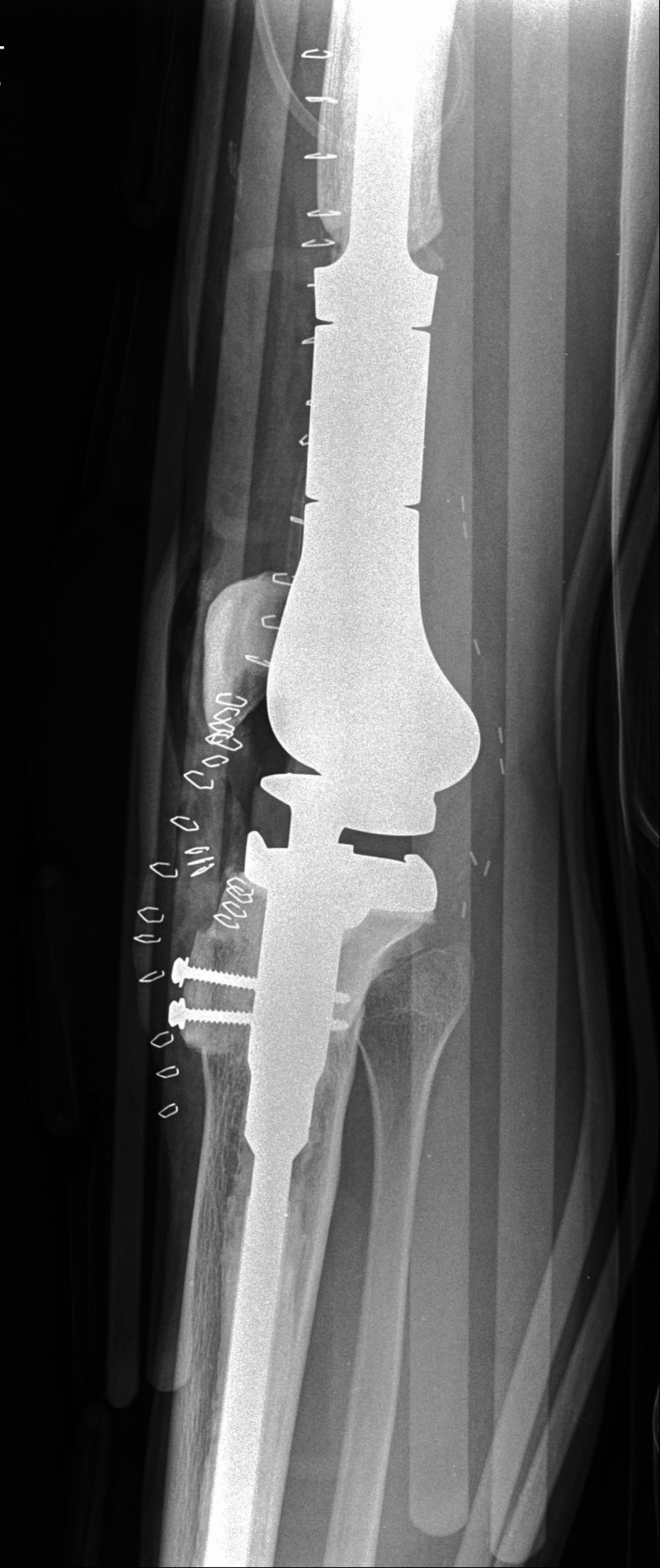
Fixation of the allograft bone block to the tibia using two screws and correction of the patella alta

Chronic patellar tendon ruptures usually require augmentation in the form of allograft, autograft, or various synthetic materials. Bone-patellar tendon-bone grafts, Achilles tendon allografts, and synthetic materials, such as polypropylene mash, have been tried to augment the repair with variable success. Figures 8-12 show a technique for repair of chronic patellar tendon rupture in a patient with distal femur replacement using bone-patellar tendon allograft. 

Synthetic material appears to be a potential alternative to allografts since they do not depend upon the availability of local muscle flaps, is more cost-effective, and yields similar results in an early study (Table 4) [25].

**Table 4:** Results of Synthetic Material Reconstruction for Patellar Tendon Dysfunction

Study	Technique	N	Results	Conclusion
Browne et al. [26]	Knitted monofilament polypropylene graft (Marlex mesh*)	13	Mean lag was less than 10° in 9 survived grafts; 3 patients failed reconstruction and needed reoperation; 1 patient ended up with arthrodesis for infection	Marlex mesh is significantly inexpensive in comparison to allograft and yields equivalent results

**Figure 13:**
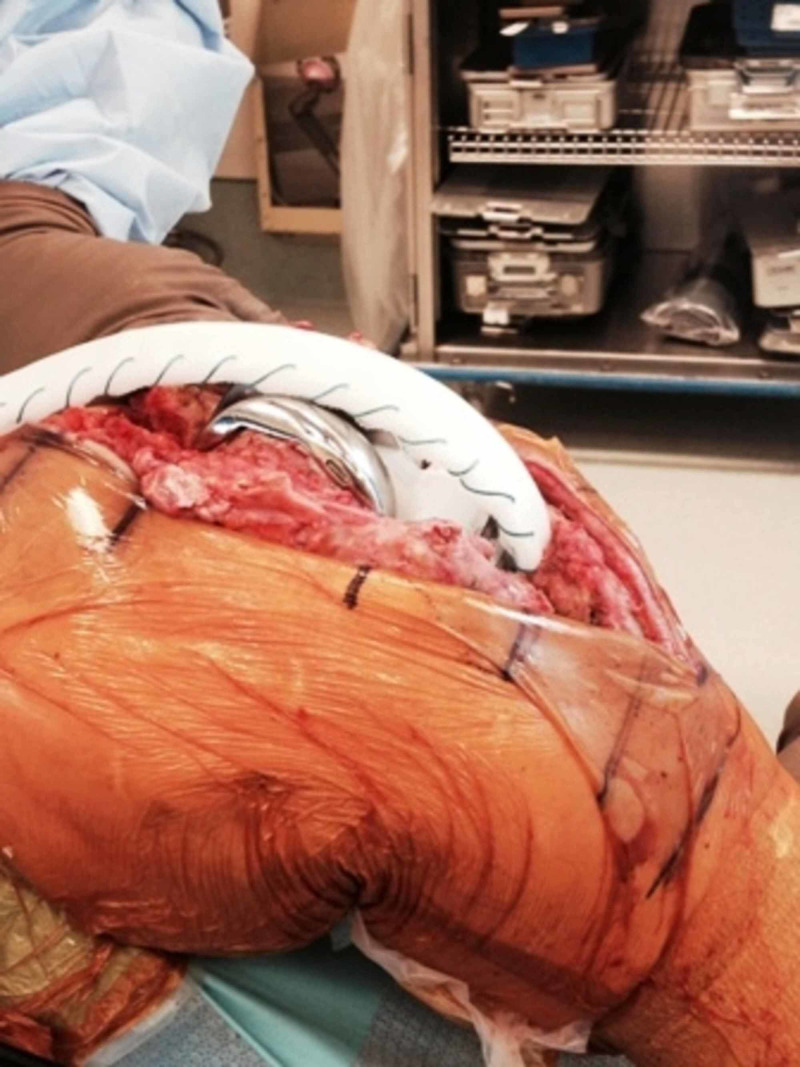
Tubularized Marlex mesh is fixed to a trough made in the proximal tibia using cement

**Figure 14:**
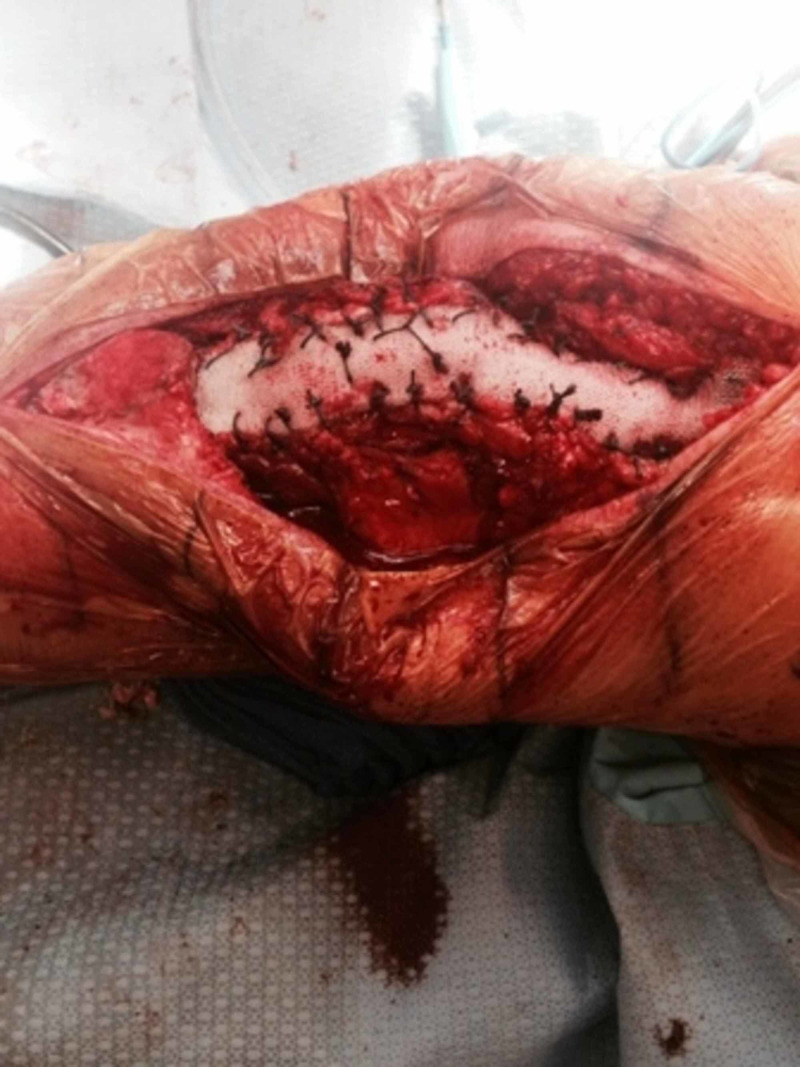
Final attachment of the Marlex mesh to the medial and lateral retinacula and quadriceps tendon with Ethibond sutures with the knee in full extension and maximum tension Ethibond sutures (Johnson & Johnson, New Brunswick, NJ)

Figures 13 and 14 show the operative technique for repair of chronic extensor mechanism disruption using the Marlex mesh (Becton, Dickinson & Co., E. Rutherford, NJ).

A chronic retracted patellar tendon with extensive scarring and soft tissue or bone loss can be treated with an extensor mechanism allograft. This procedure is technically challenging and is associated with high failure and complication rates. Some of the studies are listed in Table 5 and show results of allograft reconstruction for patellar tendon disruptions. To date, allograft reconstruction is considered the gold standard technique for chronic patellar tendon disruptions. However, on long-term follow-up, the results deteriorate because of immune reaction and resultant fibrous changes which lead to the development of late extensor lag. Better substitutes for allograft should be a focus of further results because of the high cost, risks of infection, and immune-mediated late failures.

**Table 5:** Results of allograft reconstruction for patellar tendon dysfunction HSS: Hospital for Special Surgery; N: number; pts: patients; TKA: total knee arthroplasty

Study	Technique	N	Results	Conclusion
Malhotra et al. [27]	Patella-patellar tendon-tibial tubercle composite allograft	4	One patient had a 10° lag	Provided with an optimum environment for bony healing and supervised physical therapy protocol, this technique can produce good results
Burnett et al. [28]	Quadriceps tendon-patella-patellar tendon-tibial tubercle allograft or Achilles tendon allograft	19	Mean lag 14°, 89% satisfaction rate; 15 patients had < 15° lag	Both the allografts provided satisfactory functional outcomes
Burnett et al. [29]	Quadriceps tendon-patella-patellar tendon-tibial tubercle allograft; Without tension (Group I: 7) and with tension (Group 2: 13)	20	Group I: Average extensor lag: 59° and HSS knee score: 52; Group II: Average extensor lag 4.3° and HSS knee score: 88; No loss of flexion in either group.	Repair without tension is tantamount to failure when allograft is used for reconstruction
Barrack et al. [30]	Achilles tendon allograft (N: 8); Quadriceps tendon-patella-patellar tendon-tibial tubercle allograft (N: 6)	14	Lag < 10°: 10 pts.; Lag -15° : 2 pts.; Lag - 30°: 1 pt.; Lag - 45°: 1 pt.; All patients remained ambulatory	Improved functional results and patient satisfaction with this technique
Crossett et al. [31]	Achilles tendon allograft, Group I: Primary TKA (N:5); Group II: Revision TKA (N:4)	9	Improvement in knee functional score Group I: 26 to 81; Group II: 14 to 53; Average extensor lag: 44° to 3°; 2 failures with successful repairs	Once healed, Achilles allograft can serve as a reliable reconstruction, at least at short-term
Nazarian et al. [32]	Quadriceps tendon-patella-patellar tendon-tibial tubercle allograft	40	Average lag 13°, 8 patients had a rupture and repeat reconstruction; Knee Society Score improvement: 34 to 36	Results support the use of this technique over the direct repair
Emerson et al. [33]	Extensor mechanism allograft	15	No lag: 6 patients; Graft rupture: 1 patient; Early quadriceps junction failure: 1 patient; Patellar component loosening: 1; Average postoperative clinical score: 78	Satisfactory outcomes from allograft reconstruction
Emerson et al. [34]	Quadriceps tendon-patella-patellar tendon-tibial tubercle allograft	13	Minimal extensor lag: 3 patients; Failures: 3 patients required reoperation	Satisfactory outcomes from allograft reconstruction

Management of failed allograft reconstruction is truly challenging. There is a paucity of available literature in this area. In a published study, eight cases of allograft failures were re-operated using allografts [35]. Two out of the eight cases failed due to infection and the remaining six showed no clinical improvement. Repetitive surgeries further compromise the soft tissue envelope and increase the risk of infection. In refractory cases, knee fusion or amputation may be appropriate choices. The decision should be made only after due consideration of the patient’s expectations, physiological condition, and anticipated outcomes.

## Conclusions

There are many techniques described in the literature to treat extensor mechanism discontinuity. Lack of unanimity between experts probably shows the complexity of this problem and the guarded prognosis of the condition.

In summary, non-operative treatment for partial disruptions at any anatomical location results in acceptable outcomes. For complete disruptions, treatment depends upon the location, extent, and chronicity of the disruption, the patient's overall health condition and expectations, and the experience and expertise of the operating surgeon.

Every surgeon should adopt the technique that works well in his or her hands. It is also important to discuss all the aspects of the condition (e.g., treatment options, nature of the treatment, rehabilitation, and possible outcomes) in detail with the patient to bring mutual expectations to a pragmatic level.
